# The promise of layer-specific neuroimaging for testing predictive coding theories of psychosis

**DOI:** 10.1016/j.schres.2020.10.009

**Published:** 2022-07

**Authors:** J. Haarsma, P. Kok, M. Browning

**Affiliations:** a Wellcome Centre for Human Neuroimaging, University College London, London, United Kingdom; b Department of Psychiatry, University of Oxford, Oxford, United Kingdom; c Oxford Health NHS Trust, Oxford, United Kingdom

**Keywords:** Predictive coding, Psychosis, Laminar fMRI

## Abstract

Predictive coding potentially provides an explanatory model for understanding the neurocognitive mechanisms of psychosis. It proposes that cognitive processes, such as perception and inference, are implemented by a hierarchical system, with the influence of each level being a function of the estimated precision of beliefs at that level. However, predictive coding models of psychosis are insufficiently constrained—any phenomenon can be explained in multiple ways by postulating different changes to precision at different levels of processing. One reason for the lack of constraint in these models is that the core processes are thought to be implemented by the function of specific cortical layers, and the technology to measure layer specific neural activity in humans has until recently been lacking. As a result, our ability to constrain the models with empirical data has been limited. In this review we provide a brief overview of predictive processing models of psychosis and then describe the potential for newly developed, layer specific neuroimaging techniques to test and thus constrain these models. We conclude by discussing the most promising avenues for this research as well as the technical and conceptual challenges which may limit its application.

## 1 Introduction

Symptoms of psychosis including hallucinations and delusions are associated with various psychiatric and neurological disorders, including schizophrenia, bipolar disorder, Parkinson's and Alzheimer's disease. However, understanding these symptoms and how they emerge has been challenging. One line of work, often referred to as the neuropsychiatric approach, aims to understand psychiatric symptoms in terms of alterations in normal cognition (Halligan and David, 2001). Applying this approach to delusions and hallucinations, the question then becomes how changes in learning and perceptual inference might lead to these symptoms. This question initially seems to require two separate answers, one attempting to understand delusions in terms of deficits in learning and one to understand hallucinations in terms of deficits in perceptual inference. In contrast, computational theories of brain function developed in recent decades suggest that belief and perception draw upon similar computational processes, leading to the proposal that both hallucinations and delusions may arise from a common set of processes. A particularly prominent account of psychosis which takes this view is the hierarchical predictive coding theory of psychosis (Fletcher and Frith, 2009). In brief, this theory postulates that the brain forms a hierarchical model of its environment, where each hierarchical level maintains a belief about the most likely cause of its inputs, which is updated by discrepancies between the level's prior belief and its inputs (prediction errors). According to this view, hallucinations and delusions result from aberrant neural signalling of prior beliefs, sensory input and/or prediction errors (Friston, 2005; Stephan et al., 2006; Fletcher and Frith, 2009; Adams et al., 2013; Sterzer et al., 2018).

**Fig. 1:**
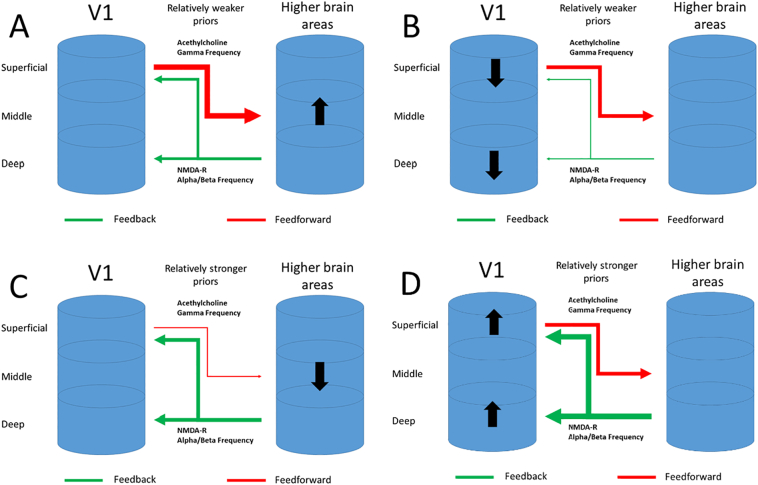
Four competing hypotheses for hierarchical disruption in psychosis. A simplified schematic representation of communication between lower levels of the cortical hierarchy (labelled as v1) and higher levels (labelled higher brain areas). Higher areas communicate their estimate of the cause of sensory events through feedback connections (“priors”, green arrows), lower areas communicate the unexplained sensory evidence through feedforward connections (red arrows). The relative influence of these connections is governed by their precision (represented as the thickness of the green/red arrows). Various predictive processing accounts of psychosis have postulated a relatively reduced influence of priors (e.g. to explain the resistance to illusions of people with psychosis) which may be caused by increased weighting of sensory evidence (panel A) or decreased weighting of priors (panel B). Other predictive processing accounts of psychosis have postulated relatively greater influence of priors (e.g. to explain increased effect of learned associations on perception in psychosis) which may be caused by reduced influence of sensory evidence (panel C) or increased influence of priors (panel D). Discrimination of these competing hypotheses requires assessment of the relative weighting of top down vs bottom up connections (the thickness of the green vs. the red arrows). Practically this requires assessment of functional activity within separate cortical laminae.

However, while predictive coding models of psychosis propose a relatively simple unifying mechanism for a range of symptoms of psychosis, this mechanism is thought to be implemented across many layers of a putative hierarchy that has not yet been well characterised. As a result the models are effectively over parameterised; they are able to explain the same behaviour in multiple ways, by postulating different perturbations at different levels of the hierarchy (Bowers and Davis, 2012; Williams et al., 2018; Litwin and Miłkowski, 2020). To complicate matters further, a given area of the cortex is thought to simultaneously implement both ascending and descending predictive coding processes (Fig. 1) in different cortical layers. Most neuroimaging techniques are unable to discriminate layer specific activity and so have been of limited use in constraining the various hypotheses generated by predictive coding accounts of psychosis.

In the present paper we will briefly summarise hierarchical predictive coding models of the brain and how they have been used to understand hallucinations and delusions. Whilst describing its application to these symptoms, we will highlight how different model variants have been used to account for the same research findings. We will then describe a novel neuroimaging technique, laminar fMRI, which is able to assess layer specific cortical activity. Finally, we will describe how this technique might help us constrain predictive coding models of psychosis, in particular by its ability to disentangle bottom-up and top-down signals, as well as describing potential limitations to the utility of this technique.

## 2 Normative predictive coding models – the theory

Before discussing how predictive coding has been used to understand hallucinations and delusions, we will first introduce the normative predictive coding model as it is commonly described. The idea that the brain aims to predict the outside world is not novel. Indeed, this idea dates back to Helmholtz who provided the crucial insight that the brain needs to infer the causes of a given sensory input, which can be achieved through combining new sensory data with pre-existing knowledge of the world (Von Helmholtz, 1867). These theories were later developed to suggest that the brain generates hypotheses about the causes of its sensory inputs (see for example Neisser, 1967; Gregory, 1980; Yuille and Kersten, 2006 for a review).

Predictive coding theories offer a set of solutions as to how this modelling of the world might be achieved, algorithmically and in terms of neural implementation (Clark, 2013, Clark, 2015; Hohwy, 2013). Predictive coding approaches have been applied to general signal processing problems (Makhoul, 1975), as well as understanding the function of specific neural structures such as the retina (Srinivasan et al., 1982). However in the present paper we focus on how predictive coding has been used to understand cortical processing and its application to psychosis (Rao & Ballard, 1999; Spratling, 2008, Spratling, 2010; Friston, 2009, Friston, 2010).

Although multiple, subtly different implementations of predictive coding have been proposed (see Spratling, 2017 for an overview), the common factor across them is that the brain forms a hierarchical generative model of its environment, where predictions are made about the causes of sensory information. These predictions are compared to sensory input, which in the case of a discrepancy creates a prediction error that is used to update the model's predictions, iteratively improving the model. Both predictions and sensory inputs are conceived of as probability distributions with a mean and variance. The mean provides an estimate of the current belief or sensory evidence whereas the variance estimates how certain this belief or evidence is. This certainty is a crucial component of predictive processing models, where it is often called “precision” (simply the inverse of the variance), and is used to weight the influence of higher level beliefs versus lower level evidence. To give an example, when sensory input is less precise, as in a rain storm where the rain drops blurs the visual field, prior predictions will tend to influence perception more. Thus, if we expect that someone is out in the rain looking for us, we will be more likely to perceive a figure standing in the rain, because our sensory inputs are less precise and have a reduced impact relative to our belief. Here it is important to point out that an identical outcome (perceiving a figure in the rain) may also be produced by increasing the precision of the prior belief rather than reducing the precision of the sensory inputs, illustrating a more general aspect of predictive processing theories—it can be difficult to discriminate reduced/increased precision at a lower level from the reciprocal change in precision at a higher level.

The brain's sensory systems are well placed to integrate prior expectations with sensory input as described by predictive processing models. The neocortex consists of six distinct layers (Felleman and van Essen, 1991), and feedback and feedforward connections between levels in the sensory hierarchy have different laminar profiles. Specifically, feedback connections preferentially originate from deep layers and terminate in agranular deep and superficial layers, avoiding the middle layers, whereas feedforward connections preferentially originate from superficial layers, and terminate in granular middle layers (see Fig. 1). Predictive coding is thought to be implemented through these connections, where predictions are conveyed by the feedback connections, and prediction errors by the feedforward connections (Felleman and van Essen, 1991; Angelucci et al., 2002; Bastos et al., 2012). This proposed laminar specificity of top-down and bottom-up processes in hierarchical predictive coding is – although biologically plausible – very difficult to test empirically in humans, as conventional neuroimaging methods cannot differentiate between cortical layers due to low spatial resolution (see Box 1 for a summary of the non-clinical evidence that hierarchical predictive coding accurately describes brain function). Therefore, without the ability to differentiate between cortical layers, predictive coding theories generally, and those related to psychosis specifically, are challenging to formally test. We argue that recent advances in neuroimaging methodology have the potential to change this (Lawrence et al., 2019a, Lawrence et al., 2019b; Stephan et al., 2019).

Box 1 **What is the evidence that the brain processes information in the way described by hierarchical predictive coding accounts?**

Hierarchical predictive coding theories make a number of explicit predictions about the neural processes we should observe, and there is varying support for these different predictions (reviewed in Walsh et al., 2020 in detail). We will briefly review these lines of evidence as they directly pertain to how predictive coding is applied in psychosis. First, neural responses to sensory stimuli should scale inversely with how predictable these stimuli are. This prediction is supported by reports of increased neural responses to unexpected stimuli, and even unexpected omissions of predicted stimuli (Heilbron et al., 2018; Grotheer et al., 2016; de Lange, 2018 for reviews). Second, top-down signals should represent sensory predictions. There is indeed evidence that expectations can induce stimulus-specific sensory signals in early sensory regions (Ekman et al., 2017; Kok et al., 2016; Aitken et al., 2020a). Third, in each level of the cortical hierarchy there are two neural populations, one signalling error and the other predictions. There is anatomical evidence that feedback and feedforward connections indeed originate from different cell populations (Markov et al., 2013; Berezovskii et al., 2011) and oscillate in different frequencies (Bastos et al., 2012; Heilbron et al., 2018; Arnal et al., 2012). Crucially, some studies report different cell populations reporting mismatch, omission and prediction signals in V1 in mice (Keller et al., 2012; Fiser et al., 2016; Attinger et al., 2017; Leinweber et al., 2017). However, evidence that prediction errors originate from distinct feedforward projecting cortical neurons, as well as evidence that predictions originate from distinct feedback projecting neurons is lacking (Walsh et al., 2020). Fourth, prediction error minimization is achieved through reciprocal exchange of error and prediction signals across hierarchical levels. This hypothesis is possibly the most difficult to test. There is indeed evidence in favour of hierarchical processing in sensory systems, but the extent to which this is in line with predictive coding accounts remains contested (Walsh et al., 2020). In summary, there is broad support for the existence of the neuroanatomical connections and cell populations in the brain required to support predictive processing and some evidence that these connections and populations propagate the information described by predictive processing accounts. However, the empirical data may also be consistent with alternative hierarchical architectures which pass different messages between layers than those specified by predictive processing models. More research will be needed to demonstrate whether or not cortical processing is indeed implemented in accordance to predictive coding theories, in which laminar fMRI is expected to play an important role (Stephan et al., 2019; Lawrence et al., 2019).

Alt-text: Box 1

## 3 Hierarchical predictive coding and how it has been applied to psychosis

Although the empirical fate of predictive coding in humans is not yet decided (see Box 1), the theory has inspired many researchers to try and understand the causes of specific psychotic symptoms in predictive coding terms. This approach has motivated various inventive studies that have sought to test predictive coding accounts of psychosis (see below). However, the dearth of direct measures of the physiological processes believed to implement predictive coding in the human brain has led to a lack of constraint on the proposed models. The first hierarchical predictive coding theories of psychosis emerged 15 years ago (Friston, 2005; Stephan et al., 2006). In these papers it was suggested that hallucinations can be conceived as a symptom that might be explained by overly *precise* priors, possibly as the result of aberrant cholinergic neurotransmission (Fig. 1d). A hallucination could arise in these circumstances in the presence of noisy sensory input. This account has face validity, as complex hallucinations are the presence of a percept in the absence of sensory input, so therefore seem to rely on strong, prediction error suppressing, top-down signals. Indeed, there is evidence suggestive of overly strong prior expectations in psychosis, with studies showing enhanced influence of often explicitly learned prior beliefs in visual (Schmack et al., 2013; Teufel et al., 2015; Davies et al., 2018) and auditory inference (Cassidy et al., 2018; Powers et al., 2017; Haarsma et al., 2020b; although see Valton et al., 2019). It should be noted that the evidence cited for overly precise priors (Fig. 1c), could equally be the product of reduced precision of sensory evidence (Fig. 1d).

Fletcher and Frith (2009) used a hierarchical predictive coding account to understand both hallucinations and delusions in terms of aberrant coding of prediction error. Specifically, these authors propose that priors are relatively *imprecise* in psychosis, which renders everything surprising, or salient, characterising delusional mood (Fig. 1b). In predictive coding terms, even predictable stimuli evoke large *prediction errors,* since they are not sufficiently explained away due to imprecise predictions. These inappropriate prediction errors in turn result in inappropriate updates of patients' model of the world, i.e., the formation of delusional beliefs. Applying this framework to auditory hallucinations, it is argued that these can arise when inner speech is misinterpreted as external sounds (Feinberg, 1978). This is thought to be the result of an aberrant efference copy, which is an internally generated neural signal that informs the brain that a sensation is internally generated (as in internal speech). When this efference copy is aberrant, the brains auditory cortex is not informed that internal speech is internally generated, resulting in an external attribution (Crapse and Sommer, 2008). This efference copy can be conceived in a predictive coding framework as a prediction that, in the context of hallucinations, is less precise. Indeed there is evidence that individuals with schizophrenia fail to attenuate the sensory consequences of their own actions (Shergill et al., 2005; Blakemore et al., 2000; Ford and Mathalon, 2004). Further evidence supporting imprecise priors in psychosis comes from studies using illusions, which rely on prior knowledge (Silverstein and Keane, 2011). For example the hollow-mask illusion, where a concave mask is perceived as convex due to the brain's strong priors that faces are convex (Gregory and Gombrich, 1973), is reduced in schizophrenia (Schneider et al., 2002) due to impaired feedback signalling (Dima et al., 2009, Dima et al., 2010). Another example is the McGurk illusion, which individuals with schizophrenia are less susceptible to (Pearl et al., 2009; White et al., 2014). It should be noted here, that the evidence for imprecise priors (Fig. 1b) could equally be the product of increased precision of sensory evidence (Fig. 1a). To further support their case for aberrant prediction errors in psychosis, Fletcher and Frith (2009) cite evidence from associative learning. In various associative learning models, prediction errors play an important part in signalling discrepancies between obtained and expected value (reward or signed prediction errors; Rescorla et al., 1972; Sutton and Barto, 1998; Schultz et al., 1997) as well as signalling the absolute difference between obtained and expected value (surprise or unsigned prediction errors) which play an important part in learning about the statistics of the environment (Pearce and Hall, 1980; Den Ouden et al., 2012; Mathys et al., 2011, Mathys et al., 2014). Both forms of prediction errors can be found in the brain (Fouragnan et al., 2018 for a meta-analysis), and both have been shown to be perturbed in psychosis (Corlett et al., 2007a; Murray et al., 2008; Ermakova et al., 2018). However, although these findings are compatible with hierarchical predictive coding, they do not address the core elements of predictive coding, i.e. scaling of prediction errors to precision, and the hierarchical nature of inference (see section above). More recently there has been some evidence for aberrant precision-weighting of prediction errors in psychosis (Haarsma et al., 2020a), as well as evidence for specific perturbations during hierarchical learning in psychosis (Cole et al., 2020). Although these studies are addressing hypotheses from predictive coding specifically, they are still limited in that they cannot discern the layer specific implementation, and therefore cannot distinguish whether aberrant prediction errors are due to impaired top-down or bottom-up signals (conflating Fig. 1a with 1b and 1c with 1d).

The disagreement with regards to whether priors are relatively more or less precise can be partially resolved by considering that different aspects of psychosis are related to more or less precise prior expectations. Adams et al. (2013) argues that psychosis can be understood as a disorder of precision. Distinguishing between state and trait abnormalities, they argue that trait abnormalities, including negative symptoms and soft neurological signs, can be understood as a relative decreased precision of priors. In contrast state abnormalities such as hallucinations and delusions can be understood as potential compensatory increase in precision of priors as previously suggested (Friston, 2005; Stephan et al., 2006). They demonstrate using simulations that the increased precision of prediction error can be counteracted by increasing the precision of the prior (panel 1c) at the cost of dissociating from the sensory environment, characterising hallucinations and delusions. In terms of the biological implementation, it is argued that NMDA receptors are well placed to encode post-synaptic precision in top-down projecting deep pyramidal cells, whereas dopamine receptors might play a role in encoding the precision of bottom-up prediction errors in superficial pyramidal cells. In short the hypothesis put forward by Adams et al. (2013) attempts to explain various symptoms of psychosis in terms of aberrant precision, with different effects occurring at different time points across the illness, and offers a neurobiological hypothesis about how it might be implemented in the brain.

The most recent iterations of the hierarchical predictive coding account of psychosis at time of writing argues for a more nuanced perspective still (Sterzer et al., 2018; Heinz et al., 2019). It is proposed that, although the brain might be summarized as performing some kind of hierarchical Bayesian inference, it does not follow that psychosis can be characterised as a gross overweighting or underweighting priors. Similar to Adams et al. (2013), it is suggested that different aspects of psychosis might be associated with more or less precise prior expectations. Sterzer et al., 2018 and Heinz et al., 2019 expand the theory by including different sensory modalities, different hierarchies, as well as different disease stages, which can all be associated with aberrant precision in prior expectations and/or sensory input in complex interacting ways (panels 1a-d).

In summary, separate, mutually exclusive, hierarchical predictive coding theories of psychosis have been proposed over the last 15 years. The individual theories are able to account for some aspects of experimental findings, but there is little strong evidence to favour one theory over another. Strikingly, most theories pertain to physiology at the laminar level of the cortex, and with the lack of appropriate neuroimaging methods, it is challenging to discriminate between the competing accounts. In the next section we introduce laminar fMRI and suggest that it may be a useful tool for adjudicating between competing predictive processing accounts of hallucinations and delusions.

## 4 Laminar fMRI: might it provide a useful test of predictive coding theories of psychosis?

Layer-specific fMRI was first described a decade ago (Koopmans et al., 2010). However, it is only recently that ultra-high field MRI scanners, which allow for the acquisition of submillimetre fMRI data, have become widely available. Various studies have demonstrated that laminar fMRI can disentangle bottom-up and top-down cognitive processes. For example, visual bottom-up sensory input has been shown to preferentially activate the middle layers of V1 (Koopmans et al., 2010; Lawrence et al., 2019b) in line with neuroanatomical studies (Felleman and van Essen, 1991; Angelucci et al., 2002). According to the same neuroanatomical studies, top-down signals should terminate in deep and superficial layers, avoiding the middle layers. Indeed a number of laminar fMRI experiments studying different top-down effects including attention, working memory and prediction have found corresponding activity dominating in deep and superficial layers (Lawrence et al., 2018; Lawrence et al., 2019a, Lawrence et al., 2019b; Klein et al., 2018; De Martino et al., 2015; Muckli et al., 2015; Gau et al., 2020; Kok et al., 2016; Aitken et al., 2020a, Aitken et al., 2020b). Thus, although the technique of laminar fMRI is still in its infancy, it is encouraging that studies like Lawrence et al., 2018, find laminar specific profiles of working memory in superficial and deep-layers but not in the middle layers, perfectly in line with physiological studies in mammals (Van Kerkoerle et al., 2017). The ability of laminar fMRI to measure layer specific top-down and bottom-up effects suggests that it is well suited to study hierarchical predictive coding theories of psychosis which pertain to the contribution of different cortical layers in signalling top-down prior expectations and bottom-up prediction errors. In the following section we provide specific examples of how laminar fMRI might be used to achieve this goal. For the sake of argument, we will assume for the remainder of this paper that prediction errors are most likely to be detected in the superficial layers of the cortex, in line with neuroanatomical studies (Felleman and van Essen, 1991, Angelucci), predictive coding theories (Bastos et al., 2012), as well as evidence demonstrating error signalling in supragranular layers (Bonaiuto et al., 2018a, Bonaiuto et al., 2018b; Sajad et al., 2019). However, it should be kept in mind that since feedforward connections arising from superficial neurons terminate in the middle layers of downstream regions (Felleman and van Essen, 1991; Angelucci et al., 2002), future studies might demonstrate that BOLD activity in the middle layers might reflect prediction errors as well, reflecting the post-synaptic input from these lower-level forward-projecting superficial pyramidal cells.

Various paradigms have been used to study how predictions differentially affect perception in psychosis. Although sometimes neuroimaging data have sometimes been collected while participants complete these paradigms, to date this has not included laminar imaging able to address the key questions described above. Starting with visual illusions, psychosis has been associated with a resistance to specific illusions, which has been framed as evidence in favour of aberrant precision of prior expectations in psychosis (Notredame et al., 2014; Keane et al., 2016), although it could in theory also be related to increased precision of sensory input (see Fig. 1a&b). Laminar fMRI can be used to study visual illusions, such as the Kanizsa illusion, for which it has been shown that illusory contours are signalled in the deep layers of V1 (Kok et al., 2016) in line with subsequent animal studies (Pak et al., 2020). Individuals with schizophrenia tend to be more resistant to various illusions such as the Kanizsa illusion (Spencer et al., 2003, Spencer et al., 2004; but also see Foxe et al., 2005). It would be possible to use the paradigm used by Kok et al. (2016), where participants undergo laminar fMRI scanning whilst viewing the Kanizsa illusion or a control stimulus. Using a retinotopic mapping procedure the regions in V1 coding for the illusory contours can be assessed directly to test whether resistance to an illusion is indeed related to aberrant feedback processes in the deep layers of V1, reflecting reduced precision of a perceptual prior (Fig. 1b). In contrast, if there is indeed precision in sensory input, this would be reflected by increased activity in superficial layers in retinotopic regions where sensory input is presented (Fig. 1a). Similarly, some studies have reported decreased susceptibility to cross-modal illusions in psychosis, including the McGurk effect (Pearl et al., 2009; White et al., 2014; Haarsma et al., 2020b), which has been interpreted as an example of predictive coding (Arnal et al., 2011; Blank and Davis, 2016). Laminar neuroimaging could similarly be used to test whether the resistance to certain cross-modal illusions is indeed due to aberrant processing in specific layers (Gau et al., 2020).

In contrast to the lower-level expectations proposed to underlie the illusions mentioned, previous studies have suggested that prior expectations induced by higher order learned associations are *stronger* in psychosis (e.g. Schmack et al., 2013; Powers et al., 2017; Cassidy et al., 2018; Haarsma et al., 2020b; however also see Valton et al., 2019). In order to test this hypothesis directly, laminar fMRI can be used in combination with tasks where the participants learns to predict the most likely sensory stimulus. Previous experiments in patients have suggested that individuals with a history of hallucinations are prone to hallucinatory experiences under these conditions due to an increased precision in priors (Powers et al., 2017). Studies in healthy individuals have demonstrated that the mere expectation of a stimulus induces activity in the deep layers of V1 which is specific to the expected stimulus (Aitken et al., 2020a). If it is indeed the case that conditioned hallucinations as in Powers et al. (2017) are due to more precise priors, this should be reflected by increased activity in the deep layers of V1 specific to the hallucinated stimulus (Fig. 1c). Alternatively, the relative increase in prior expectations might be the result of decreased precision in sensory input. This could be tested by studying the activity induced by sensory stimulation. If psychosis is characterised by a reduction in precision of sensory input, this would be reflected in diminished activity in the superficial layers during sensory stimulation. Furthermore, the increased precision of prior expectations might be secondary to an initial increase in the precision of bottom-up prediction errors during the learning stage of the experiment where the association between a cue and a stimulus is learned. This question remains unexplored as participants are often assumed to have learned the association between a cue and a stimulus equally well before the experiment is started. However, if the learning stage of the experiment were to be included in the imaging phase of the experiment, we might find that individuals with a history of hallucinations are characterised by *increased* signalling of prediction error in the superficial layers of V1 during learning. This could result in an overly precise prior during the experimental phase of the experiment, as reflected by increased expectation induced activity in the deep layers of V1. In this way hallucinations might be the result of *increased* precision of prediction error as well as *increased* precision of prior expectations at different times in the same experimental paradigm.

So far, we have described how laminar fMRI can directly test the hypotheses that psychosis is associated with increased/decreased signalling of prior expectations or whether alternative mechanisms are responsible (e.g., lowered precision of sensory input). However, in order to establish whether psychosis is the result of *specifically* hierarchical predictive coding effects, a further four hypotheses need to be tested. Here we refer back to the four specific hypotheses that follow from normative hierarchical predictive coding theory (see Box 1 & Walsh et al., 2020). The first hypothesis is that psychosis is characterised specifically by aberrancies in the coding of precision, in particular the scaling of prediction errors in proportion to their precision (Adams et al., 2013). There is some evidence for alterations in the role of precision in perceptual inference in psychosis (Cassidy et al., 2018), as well as precision-weighting of prediction error (Haarsma et al., 2020a). In order to test this hypothesis directly, paradigms will be needed which vary the precision of sensory input (for example by introducing noise in sensory stimuli) as well as the precision of prior expectations (for example by varying the predictability of cue-outcome relationships in sensory conditioning). Using these paradigms it is possible to explore whether prediction errors are indeed scaled to precision in the superficial layers, and whether this is indeed aberrant in psychosis. Crucially, in order to disentangle prediction errors from sensory input, paradigms must be used where the predictability of sensory input is varied. If superficial layers indeed reflect prediction error, activity must be observed to reduce as the predictability of the stimulus increases. Indeed, when sensory input is unpredicted, prediction errors will be identical to sensory input, and are therefore inseparable from each other, and thus varying predictability is key. In other words, prediction errors are supposed to reflect sensory input with predictions subtracted out. Second, the hierarchical predictive coding theory of psychosis makes the prediction that feedback connections convey predictions. If enhanced activity in deep layers is demonstrated in psychosis, potentially reflecting stronger prior expectations, it will need to be demonstrated that this feedback activity reflects stimulus-specific predictions by decoding the content of these signals, as has been done using laminar fMRI in humans (Aitken et al., 2020a). This will provide support for the view that feedback activity reflects predictions. Third, hierarchical predictive coding postulates different cell populations conveying predictions and error signals. With respect to its application to psychosis, because the theory proposes that psychosis is associated with aberrant signalling of prediction errors, it will be important to demonstrate that possible aberrant activity in the superficial layers of sensory cortices indeed reflects aberrant coding of prediction errors, rather than simply reflecting sensory input. Finding merely aberrant coding of sensory input in the superficial layers will not be sufficient support for a predictive coding deficit in psychosis. Fourth, in order to specifically support the hierarchical predictive coding account of psychosis, it will need to be demonstrated that the alterations related to psychosis are indeed hierarchical in nature. Various studies have argued that low-level sensory context independent predictions and high-level learned context dependent predictions might be differently affected in psychosis (Sterzer et al., 2018; Heinz et al., 2019; Haarsma et al., 2020b), which might explain why some studies find stronger and others weaker priors. Laminar fMRI may be used to study whether different forms of predictions originate from different levels of the hierarchy as well as demonstrating they are differently affected in psychosis. For example, the visual system is biased towards previously presented stimuli, an effect known as serial dependence. This is believed to be a low-level perceptual effect (Fischer and Whitney, 2014; St. John-Saaltink et al., 2016), which could be driven by top-down activity. As lower-level priors have been suggested to be weaker in psychosis (Schmack et al., 2013; Sterzer et al., 2018; Heinz et al., 2019; Haarsma et al., 2020b), this might reflect a weakened effect of prior expectations in the deep layers of V1, or in contrast might reflect enhanced activity in the superficial layers of V1. In contrast, similar perceptual biases can be induced through higher-level associative learning (Aitken et al., 2020b), which might reflect expectations originating from high-level areas like the hippocampus (Hindy et al., 2016, Schapiro et al., 2012, Kok and Turk-Browne, 2018, Kok et al., 2020). Experimental paradigms that manipulate whether expectations are low-level, like serial dependence, or high-level, as when perceptual biases are induced by learned cues, in combination with laminar fMRI to investigate whether the observed effects should be attributed to altered feedback or feedforward signalling, will provide important evidence that predictive coding deficits in psychosis are indeed hierarchical in nature. Of course, it is possible that laminar fMRI itself will provide important information on foundational questions, such as the representational content of the different levels of the informational processing hierarchy. For example, by combining computational models (as in Weber et al., 2020) with laminar fMRI we could start to explore whether lower-level and higher-level prediction errors indeed map onto different levels of the superficial layers of cortical hierarchy and explore how they are perturbed in psychosis. Furthermore, by using connectivity analysis it would be possible to establish the anatomical network of lower level regions that pass prediction errors to a higher region, allowing for the characterisation of the informational nature of the prediction and prediction error represented by different levels in the cortical hierarchy.

Lastly, it should be noted that psychosis is likely to be a heterogeneous phenomenon, where similar experiences might result from stronger prior expectations at a particular level of the hierarchy in one group of individuals and weaker prior expectations at another level in other individuals. Indeed, the methods outlined above could in principle help differentiate these putative populations of patients on the basis of their neurological profiles.

## 5 What laminar neuroimaging can tell us about the neuromodulatory systems involved in encoding precision

Hierarchical predictive coding theories of psychosis suggest that different neurotransmitters are involved in signalling precision of prior expectations and prediction error. Indeed, they suggest that perturbations of these neurotransmitter systems play an important role in the emergence of psychotic symptoms (Adams et al., 2013; Sterzer et al., 2018; Heinz et al., 2019). In order to understand the neuromodulatory systems involved in encoding the precision of prior expectations and sensory evidence on the laminar level, pharmacological manipulations may be used to selectively target specific modulatory neurotransmitter systems. For example, NMDA-receptors are present in feedback pyramidal cells (Fox et al., 1989; Rosier et al., 1993) and are therefore ideally located for encoding the precision of prior beliefs. Disturbing the function of the NMDA-receptors using ketamine induces some symptoms of psychosis, in particular delusions and dissociative experiences, more so than hallucinations (Corlett et al., 2007b; Corlett et al., 2016). Because ketamine predominantly effects delusions rather than hallucinations, this might suggest that ketamine is more effective in disturbing precision in higher levels of the cortical hierarchy. Indeed, there is evidence from EEG that prediction errors about higher-level statistical regularities are particularly suppressed by ketamine (Weber et al., 2020). Laminar fMRI could be used to test whether these perturbances are due to directly affecting the coding of prediction errors in the superficial layers or whether this is secondary to altered feedback signalling in the deep layers, as would be predicted by the prevalence of NMDA-receptors on feedback neurons (Fox et al., 1989; Rosier et al., 1993). In contrast, acetylcholine has been related to stronger signalling of bottom-up information in auditory mismatch paradigms (Baldeweg et al., 2006; Moran et al., 2013. This could potentially reflect increased precision of prediction errors in lower levels of the cortical hierarchy, or weakened feedback from higher levels. In order to test this hypothesis, laminar fMRI can be used in combination with a mismatch paradigm to demonstrate that acethylcholine indeed increases signalling of bottom-up prediction errors in sensory superficial layers, or whether this is secondary due to diminished feedback activity in sensory deep layers. Inversely, scopolamine, an acetylcholine antagonist, is known to induce hallucinations by sensory conditioning (Warburton et al., 1985). This might be achieved by reducing the precision of sensory prediction errors in the superficial layers of sensory cortices, thereby decoupling the sensorium from sensory input, allowing hallucinations to manifest, rather than affecting feedback activity in the deep layers per se. Thus NMDA-receptors and the cholinergic system have been suggested to affect precision in different ways, where ketamine diminishes high-level prediction errors and acetylcholine increases low-level prediction errors. A first stage in assessing these mechanisms would involve characterising the effects of these agents on layer specific activity using laminar imaging.

## 6 Challenges to laminar fMRI

There are a number of technical challenges that need to be overcome for laminar fMRI to be used widely in psychiatric research. For example, large draining veins in the cortical surface cause a spatial bias towards the surface (Uludağ and Blinder, 2018). This complicates the interpretation of individual layer activity when using conventional bold methods (Kay et al., 2019). Alternative non-BOLD fMRI sequences (reviewed in Huber et al., 2019), such as the CBV-weighted VASO (Lu et al., 2003) method, have been shown to be locally more specific and have contrast that is more evenly weighted across the cortical depths, but with reduced sensitivity compared to Gradient Echo (GE) EPI (Huber et al., 2015). Further issues relate to the low sensitivity and restricted brain coverage, as well as motion artefacts that restrict laminar analyses that use high field strengths. However, recent developments have demonstrated that these limitations may be overcome, making laminar fMRI more suitable for widespread use (Huber et al., 2020). For example, SS-SI-VASO or MAGEC-VASO sequences provide superior brain-coverage over conventional methods, whilst not relying on the BOLD effect, thereby side-stepping the issues regarding large draining veins. With regards to motion-distortion, prospective motion-correction can reduce the amount of correction that needs to be performed post-data acquisition (Bause et al., 2020). Thus, although laminar fMRI comes with many technical challenges, the field is moving quickly to overcome these limitations.

Currently it is common practice for laminar fMRI to have a resolution of around 0.8 mm isotropic. This resolution is sufficiently high for studies of the visual cortex (which is 2.5 mm thick on average) to separate superficial (I to III) from middle (IV) and deep (V and VI) layers, as these occupy approximately one third each of the volume of human V1 and V2 (de Sousa et al., 2010). However, higher resolutions of 0.2 mm seem obtainable in future, possibly allowing the study of the individual cortical layers (Morgan et al., 2020).

By its nature, laminar fMRI seeks to measure precisely anatomically localised activity within cortical layers. This specificity can be bought by either increasing the scan time and/or reducing the regions of the brain covered by the scan. However, imaging the full cortical hierarchy implicated in psychosis is likely to require brain coverage from the visual to prefrontal cortices, and there are clearly limits on acceptable scan lengths, particularly in clinical populations. It will therefore be challenging, using currently available sequences, to provide complete assessment across this hierarchy in patient groups, and more targeted assessments, perhaps in analogue populations may well be required. However, recent studies have showed promising results demonstrating whole brain coverage of laminar fMRI sequences, which suggest these methods might be deployed in the future to study whole brain laminar interactions between high-level and low-level regions (Sharoh et al., 2019).

fMRI methodology will also likely remain limited by relatively poor temporal resolution. This is a serious limitation to consider with regards to its ability to test predictive coding theories, as these theories make specific predictions regarding the timing of specific neural effects. That is, if psychosis is associated with aberrant predictions, these should be found in pre-stimulus effects (as in for example: Alilović et al., 2019; Jabar et al., 2017; Aru et al., 2016; Kok et al., 2017), rather than post-stimulus effects which might reflect effects related to the decision stage instead (as in for example: Rungratsameetaweemana et al., 2018; Bang and Rahnev, 2017). However, recent methodological advances have made it possible to use MEG to make inferences about the contribution of different cortical layers in perceptual inference (Bonaiuto et al., 2018a, Bonaiuto et al., 2018b; Liuzzi et al., 2017, Meyer et al., 2017; Troebinger et al., 2014a, Troebinger et al., 2014b). Laminar MEG might therefore be an exciting tool to test hierarchical predictive coding theories of psychosis, as the MEG signal has millisecond temporal resolution, represents a direct measure of neural activity, and is therefore only limited by data quality and the models used to explain the data (Bonaiuto et al., 2018a, Bonaiuto et al., 2018b).

A second set of challenges to the utility of laminar fMRI in testing predictive coding models of psychosis arises from ambiguity about the neuroanatomy and functional organisation of the putative hierarchical levels of processing, particularly those outside the sensory cortices (Williams, 2018). While visual and auditory cortices are arranged in a relatively well described hierarchy which lends itself to predictive processing accounts (Felleman and van Essen, 1991), outside of sensory cortex it is less clear what levels of hierarchy exist, where and how they are instantiated and what type of beliefs they maintain. This issue would seem to be particularly pertinent to delusions; for example, how does the brain represent the belief that it is being persecuted? What counts as lower level evidence for or against this and how is that evidence represented? In the absence of a robust account of this higher level processing laminar fMRI may be more able to speak to perceptual symptoms such as hallucinations than delusions.

## 7 Conclusion

In summary, hierarchical predictive coding has provided researchers with a framework to try to understand psychotic symptoms. This framework has been helpful in understanding past experiments and driving novel research. However, predictive processing theories of psychosis have become increasingly flexible, describing various levels of prior expectations which can be separately affected in different modalities, hierarchical levels, and disease stages (Adams et al., 2013; Sterzer et al., 2018; Heinz et al., 2019). This flexibility makes the models challenging to falsify (Williams et al., 2018; Litwin and Miłkowski, 2020). We have argued here that since hierarchical predictive coding models speak to the roles that different cortical layers play, particularly in perceptual inference, neuroimaging methods that can disentangle the cortical layers are essential in testing and constraining these models. We believe that layer-specific fMRI has the potential to bring a new level of scrutiny to the field, allowing us to increase our understanding of the mechanisms underlying psychotic illness.

## Funding

PK is supported by a Sir Henry Dale Fellowship jointly funded by the 10.13039/100010269Wellcome Trust and the 10.13039/501100000288Royal Society (218535/Z/19/Z). MB is supported by a Clinician Scientist Fellowship from the MRC (MR/N008103/1) and by the 10.13039/501100013373NIHR Oxford Health Biomedical Research Centre.

## CRediT authorship contribution statement

JH, MB & PK conceived of the idea and wrote the paper.

## Declaration of competing interest

MB has received travel expenses from Lundbeck for attending conferences and acted as a consultant for Jansen Research and CHDR.
